# Cell-Penetrating Peptides: A Comparative Study on Lipid Affinity and Cargo Delivery Properties

**DOI:** 10.3390/ph3041045

**Published:** 2010-03-30

**Authors:** Paolo Ruzza, Barbara Biondi, Anna Marchiani, Nicola Antolini, Andrea Calderan

**Affiliations:** Institute of Biomolecular Chemistry of CNR, Padova Unit, and Department of Chemical Sciences, University of Padova, via Marzolo 1, I-35131 Padova, Italy

**Keywords:** antennapedia, kFGF, Tat, poly-arginine peptides, conformational studies, lipid affinity

## Abstract

A growing number of natural and/or synthetic peptides with cell membrane penetrating capability have been identified and described in the past years. These molecules have been considered promising tools for delivering bioactive compounds into various cell types. Although the mechanism of uptake is still unclear, it is reasonable to assume that the relative contribute of each proposed mechanism could differ for the same peptide, depending on experimental protocol and cargo molecule composition. In this work we try to connect the capability to interact with model lipid membrane and structural and chemical characteristics of CPPs in order to obtain a biophysical classification that predicts the behavior of CPP-cargo molecules in cell systems. Indeed, the binding with cell membrane is one of the primary step in the interaction of CPPs with cells, and consequently the studies on model membrane could become important for understanding peptide-membrane interaction on a molecular level, explaining how CPPs may translocate a membrane without destroying it and how this interactions come into play in shuttling CPPs via different routes with different efficiency. We analyzed by CD and fluorescence spectroscopies the binding properties of six different CPPs (kFGF, Nle^54^-Antp and Tat derived peptides, and oligoarginine peptides containing 6, 8 or 10 residues) in absence or presence of the same cargo peptide (the 392-401pTyr^396^ fragment of HS1 protein). The phospholipid binding properties were correlated to the conformational and chemical characteristics of peptides, as well as to the cell penetrating properties of the CPP-cargo conjugates. Results show that even if certain physico-chemical properties (conformation, positive charge) govern CPP capability to interact with the model membrane, these cannot fully explain cell-permeability properties.

## 1. Introduction

The process of introducing biological active molecules into cells, to interact with intracellular targets, has proved a major challenge for researchers and the pharmaceutical industry. The inability of peptides and oligonucleotides to cross membranes has been one of the principal obstacles to their use as tools to study cellular processes and in the development of drug candidates [1,2]. Over the last two decades a growing number of natural or synthetic peptides have been discovered with cell membrane penetranting capability and considered as promising tools for delivering bioactive compounds, which are poorly internalized by themselves, into various cell types (an exhaustive discussion can be found in [3]).

Several of these cell-penetrating peptides (CPPs) correspond to protein fragments representing either the basic region derived from the corresponding RNA-/DNA-binding domain or the hydrophobic core (h-region) of the signal sequence region. This last CPP is represented by the peptide corresponding to the sequence 7-22 of the fibroblast growth factor of the Kaposi’s sarcoma, named kFGF [4,5,6].

The HIV-1 Tat peptide sequence is an example of a CPP originated from protein basic regions. Originally the full length protein evidenced the ability to cross plasma membrane and subsequently small fragments, which efficiently enter cells, have been discovered. Through mutagenesis studies it was found that Tat penetrating capability resides in the fragment 48-60 [7,8].

Another basic peptide is represented by the third helix of the homeodomain of the Antennapedia transcription factor. The internalization property of this domain was established during studies on the neural development of Drosophila in 1991, in particular the region comprising residues 43-58, corresponding to the third helix, has been identified as responsible of penetration capability [9,10].

Additional CPPs, such as oligoarginines, have been designed, based on studies recognizing the importance of positively charged residues, and in particular of the guanido group, to mediate transport across cell membrane [11]. Indeed, although CPPs show a highly variable structure, some general features, as an abundance of positive charges, especially from arginine residues, and the presence of bulky side-chain hydrophobic amino acids, have been suggested to be essential for an efficient cellular uptake [12].

Despite a general acceptance of these molecules as vectors, the mechanism of cellular internalization and membrane permeation is still in debate. Various uptake mechanisms seem to be active in different systems and sometimes the mechanism results to be dependent on cell-type or cargo. Two major routes govern the access of CPPs to cell: endocytosis, an energy dependent vesicular mechanism, in which extracellular molecules are incorporated in lipid vesicles which are internalized; and a second route which proceed through direct translocation, in this case CPPs cross the membrane bilayer in an energy-independent process [2]. The most shared hypothesis proposes that translocation occurs via endocytosis or macropinocytosis followed by partial escape from the endocytotic vesicles of lysosomes. It is now clear that the uptake of Tat and oligoarginine, as well as that of SynB5 and Antp, proceeds mainly by an endocytic pathway rather than temperature-independent translocation [13]. On the other hand, the uptake of Tat and oligoarginines under endocytosis-blocking conditions was demonstrated recently [13], suggesting that cell-penetrating peptides are internalized by more than one mechanism, sometimes even in parallel. It is thus reasonable to assume that the relative contribution of each mechanism could differ, depending on the experimental protocol and on the nature of cargo molecules. It is possible that the ability to disturb the membrane, or the capability to bind to and thereby recruit specific membrane components, is an important event to start up the endocytotic machinery. Consequently, the studies of CPPs in model membrane could become fundamental for understanding peptide-membrane interaction on a molecular level, explaining how CPPs may translocate a membrane without destroying it and how peptide-membrane interactions come into play in shuttling CPPs via different routes with different efficiency.

**Table 1:** Names and sequences of the investigated peptides.

Nr.	Peptide	Sequence
**1**	**KFGF**	^7^AAVALLPAVLLALLAP^22^
**1a**	**kFGF-HS1pY**	AAVALLPAVLLALLAP*PEGDpYEEVLE*
**2**	**Nle^54^-Antp**	^43^RQIKIWFQNRR-Nle-KWKK^58^
**2a**	**Nle^54^-Antp-HS1pY**	RQIKIWFQNRR-Nle-KWKK*PEGDpYEEVLE*
**3**	**Tat**	^48^GRKKRRQRRRPPQG^61^
**3a**	**Tat-HS1pY **	GRKKRRQRRRPPQG*PEGDpYEEVLE*
**4**	**R6**	RRRRRR
**4a**	**R6-HS1pY **	RRRRRR*PEGDpYEEVLE*
**5**	**R8**	RRRRRRRR
**5a**	**R8-HS1pY**	RRRRRRRR*PEGDpYEEVLE*
**6**	**R10**	RRRRRRRRRR
**6a**	**R10-HS1pY**	RRRRRRRRRR*PEGDpYEEVLE*
	**HS1pY**	^392^ *PEGDpYEEVLE* ^401^

The aim of these studies is settled to clarify the structure-activity relationship for the interaction of peptides with membranes, try to identify the role of conformation on the interaction with lipid bilayers and the effect of cargo on it, besides the influence of lipid composition on peptide-lipid interactions and translocation [14] in order to obtain a biophysical classification that could predict the behavior in cell systems. Using both fluorescence and circular dichroism (CD) spectroscopies we studied the interaction and the conformation of different CPPs (Table 1) and their constructs with a phosphotyrosine containing peptide derived from the hematopoietic lineage cell-specific protein 1 (HS1) protein [15], with small unilamellar vesicles (SUVs) of different charge density.

## 2. Results and Discussion

In the overwhelming body of literature on peptides or proteins able to cross biological membranes and to promote the delivery of drugs into cells, few studies have been reported in which different vectors have been applied to the same delivery problem and, in particular, on the correlation between conformation and internalization properties. In this paper, we estimated the ability of different CPPs to interact with simplified model membranes to obtain information, on a molecular level, on CPP-lipid interactions by complementary methods based on the fluorescent properties of carboxyfluoresceinyl-labeled derivatives and on the chiroptical properties of peptides. The variables addressed in this study comprise charge and composition of the membrane-mimicking environment in addition to the vector composition. In particular, to investigate the specificity of peptides towards lipid vesicles, depending on lipid head groups, anionic and zwitterionic lipids were used.

The ability of kFGF, modified Nle^54^-Antp, Tat, and oligoarginine peptides to deliver into cell has been demonstrated by different authors [4,5,6,7,8,9,10,11]. However, the application of these CPPs to the same cargo and its influence on lipid-interactions has not been completely elucidated. In this work, we used as cargo the phosphopeptide, corresponding to the sequence 392-401pTyr^396^ of the HS1 protein (HS1pY), which previously we demonstrated to be a potent inhibitor of the secondary HS1 phosphorylation, acting at level of the Src-homology 2 domains (SH2) mediate protein recruitment [10,14].

### 2.1. Peptide binding to phospholipid

**Figure 1:**
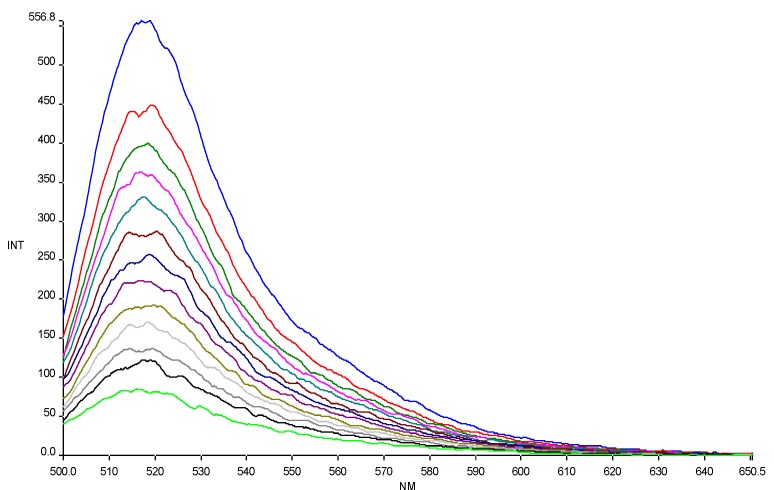
Quenching of carboxyfluorescein fluorescence emission in peptide **6a** by DMPG SUVs (15.0 mM) titration. Excitation wavelength 485 nm. Peptide 0.3 µM in 5 mM Tris-HCl buffer, pH 6.8. Phospholipid/peptide molar ratio ranging from 0.00 to 1.57.

The affinity of CPPs, alone and conjugated to HS1pY, towards phospholipid vesicles was assessed by titration of carboxyfluoresceinyl-labeled peptide solutions with liposomes of different composition (an example of titration is reported in Figure 1). As shown in Figure 1, carboxyfluorescein emission is sensitive to environment change, showing a quenching when labeled peptides are in a more hydrophobic environment after addition of phospholipids [16]. The fluorescence quenching was used to generate the binding isotherm of the labeled peptides and then to calculate the related partition coefficient (K_p_).

**Figure 2:**
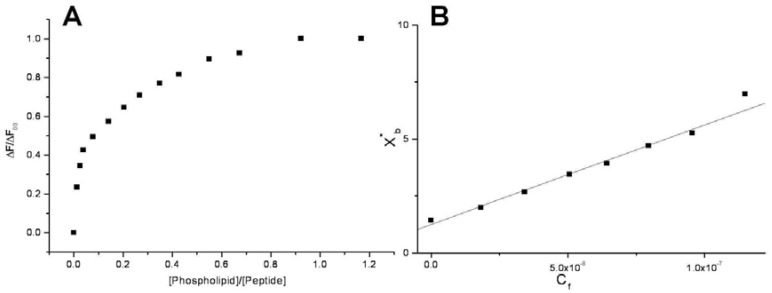
Saturation (A) and binding isotherm (B) curves of peptide **6a** (0.3 µM) titrated by DMPG SUVs (15.0 mM) in 5 mM Tris-HCl buffer, pH 6.8. Calculated K_p_ is 1.5x10^9^, with an R value of 0.995 for the linear fit. Reported values represent means of three separate experiments.

**Figure 3:**
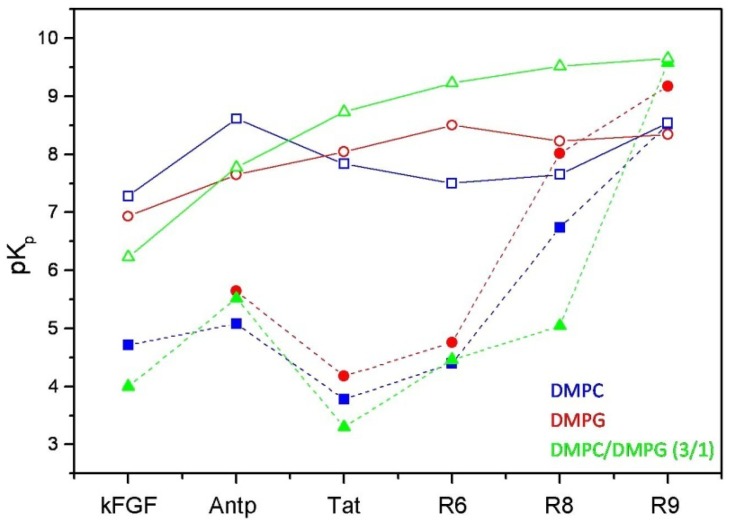
Comparison between the logarithmic values of the surface partition coefficients (pK_p_) of CPPs and corresponding conjugated peptides. CPPs are represented by open symbols, meanwhile CPP-HS1pY peptides are represented by solid symbols.

Conventional binding curves were obtained by plotting the ΔF/ΔF_∞_ as a function of lipid-to-peptide molar ratio (Figure 2A). Assuming that the peptides were initially portioned only over the outer leaflet of SUVs, conventional binding isotherm (Figure 2B) was constructed plotting the correct molar ratio of bound peptide per 60% of the total lipid (X_b_*) vs. the equilibrium concentration of free peptide in the solution (C_f_). The surface partition coefficients K_p_ are estimated by extrapolating the initial slopes of the curves to zero C_f_ values and the resulted values are summarized in Table 2 and in Figure 3.

The calculated values (Table 2) confirm the strong dependence of the peptide-lipid interactions on the nature and composition of both vesicles and peptides. As shown in Figure 3, reporting the logarithmic values of K_p_ of respective peptides, a first distinctive feature of un-conjugated CPPs is their different selectivity towards neutral (zwitterionic) or negatively charged SUVs. Taking into account of this behavior, CPPs may be separated into two groups, interconnecting different CPP families. kFGF and Nle^54^-Antp show an high affinity towards DMPC SUVs, meanwhile Tat and oligoarginines show an high affinity towards mixed DMPC/DMPG (3:1 molar ratio) SUVs, and this behavior is strongly related to the number (R6 < R8 < R10) of arginine residues.

**Table 2:** Surface partition coefficients (K_p_) of the investigated peptides in SUVs of different composition. The standard errors are less than 5% (n.d.: not determined).

Peptide	p*I*	DMPC	DMPG	DMPC/DMPG
**1**	5.57	1.9 × 10^7^	8.6 × 10^6^	1.7 × 10^6^
**1a**	2.11	5.2 × 10^4^	n.d.	1.0 × 10^4^
**2**	12.31	4.1 × 10^8^	4.5 × 10^7^	6.0 × 10^7^
**2a**	7.06	1.2 × 10^5^	4.4 × 10^5^	3.3 × 10^5^
**3**	12.70	6.9 × 10^7^	1.1 × 10^8^	5.4 × 10^8^
**3a**	8.83	6.0 × 10^3^	1.5 × 10^4^	2.0 × 10^3^
**4**	12.70	3.2 × 10^7^	3.2 × 10^8^	1.7 × 10^9^
**4a**	5.63	2.5 × 10^4^	5.7 × 10^4^	2.9 × 10^4^
**5**	12.85	6.6 × 10^7^	1.7 × 10^8^	3.3 × 10^9^
**5a**	9.55	5.5 × 10^6^	1.1 × 10^8^	1.1 × 10^6^
**6**	12.95	3.5 × 10^8^	2.2 × 10^8^	4.5 × 10^9^
**6a**	11.10	3.3 × 10^8^	1.5 × 10^9^	3.8 × 10^9^

The relationship between K_p_ constants and peptide composition can be expressed taking into account of the peptide p*I* values (Table 2). Apparently, peptides 2-6, characterized by very similar p*I* values, show a linear deviation of K_p_ values in presence of negatively charged DMPG or DMPC/DMPG SUVs. Interestingly, the K_p_ values of Nle^54^-Antp (K_p_ DMPC > K_p_ mixed > K_p_ DMPG), as well as the comparison of the K_p_ values of Tat and R6, having the same p*I* (12.70), show the influence of hydrophobic residues in addition to the positively charged ones in the binding process.

These results fit with the data reported by Liu and Deber [17], and may be summarized as follows: hydrophobic peptides, i.e. kFGF, bind and insert into lipid vesicles predominantly via hydrophobic interaction between the lipid fatty acid acyl chains and the hydrophobic peptide segment, showing high affinity towards DMPC vesicles. On the other hand, the insertion into phospholipid vesicles of peptides with low hydrophobic character may be viewed as a two step process, in which the primary electrostatic attraction (between the anionic lipid heads and the cationic side-chains of basic residues) is essential for the binding process, driving the peptides to the membrane surface. Subsequently, the hydrophobic interaction with the lipid acyl chains stabilizes the peptide-membrane interaction and affects peptide insertion.

The analysis of the binding isotherms by means of which the K_p_ were determined, provides useful information on the organization of the peptide within the membrane. Isotherm shapes are almost straight lines, suggesting a simple partition process. They differ from those observed for several pore-forming polypeptides, for which initially the curves are flat, but then their slopes rise sharply (about 100 fold) once a threshold concentration is achieved [18].

The conjugation with HS1pY strongly altered both conformational and binding properties of the tested CPPs. The remarkable K_p_ differences between vector and HS1pY conjugated peptides are mainly related to the electrostatic repulsion among the negative charges of HS1pY side-chains and the surface charges of phospholipid vesicles, confirming the important role played by electrostatic forces in the interaction of peptides with phospholipid membranes. In particular kFGF-HS1pY did not interact with DMPG vesicles, and the K_p_ determined in the presence of mixed SUVs is about one half of that of the parent peptide **1**. This behavior is shared by the other CPPs (see Table 2 and Figure 3), in particular is noteworthy the low affinity of the Tat peptide **2a**. On the other hand, the positive effect on lipid affinity of the high number of arginine residues is also highlighted by the K_p_ of the R10 derivative (**6a**): only for this peptide the K_p_ values of free and conjugated form are comparable.

### 2.2. Membrane Permeability Induced by Peptides

The efficiency of cell-penetrating peptides and corresponding HS1pY conjugates to perturb the lipid packing and causing leakage of vesicular contents has been examined. Increasing amount of selected peptide was added to mixed DMPC/DMPG vesicles containing the fluorescent dye calcein. An increase in fluorescence intensity due to the relief from the self-quenching of the calcein molecule concentrated (70 mM) within SUVs indicates the dye release. 

**Figure 4:**
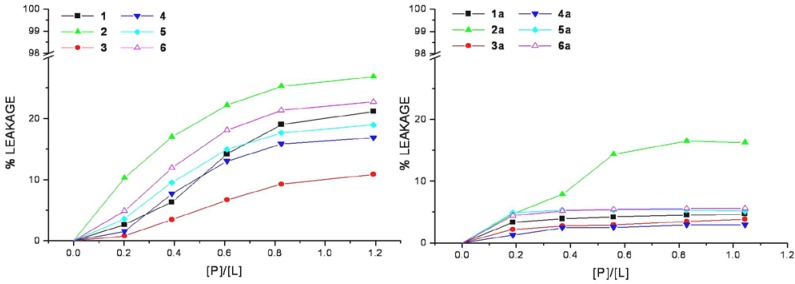
Membrane permeabilization induced by peptides. The level of membrane permeabilization was estimated by the percent of leakage of calcein from mixed DMPC/DMPG SUVs, in Tris-HCl buffer 20 mM, NaCl 150 mM, and EDTA 1 mM, pH 7.4, at 25 °C.

The relation between the apparent percent of leakage and the peptide-to-lipid molar ratio was reported in Figure 4 and indicated that none of the CPPs induces a significant leakage of dye from vesicles. In the same way even at very high peptide/lipid molar ratio (>1), the corresponding conjugates cannot significantly permeabilize vesicles as indicated by a low percentage of leakage (<20%).

Peptide-induced leakage of calcein entrapped in vesicles can be used to monitor membrane perturbation related to peptide toxicity. As shown in Figure 4, all the selected CPPs are nontoxic even at extremely elevated peptide-to-lipid ratios causing no or little calcein leakage. This property remains unaltered in the corresponding conjugated peptides, rather they show a low lytic capability in comparison to parent peptides, this may be related to the decrease of lipid affinity.

These data furnish useful information on the peptide-lipid interactions in the context of the interaction models that currently exist. In agreement with the low-lytic properties of peptides in vesicle systems and with the data obtained from the binding isotherms, it is possible to assert that the tested peptides do not form pores in the manner of certain antimicrobial peptides. Consequently, our data do not support the “carpet” and the “barrel-stave” mechanism models both inducing pore-formation [18,19].

### 2.3. Monitoring the Secondary Structure of Peptides by Circular Dichroism.

The conformational state of cell-penetrating peptides and their HS1pY conjugates in membrane mimicking environments and bound to phospholipid vesicles has been studied by CD spectroscopy.

In aqueous buffer solution the CD spectra of free CPPs were characterized by the absence of a dominating secondary structure at room temperature. On the other hand, at low temperature Tat and oligoarginine (peptides **3 – 6**) exhibited a CD pattern that closely resembles that of an extended left-handed polyproline II (PP_II_) helix conformation [20,21]. 

**Figure 5:**
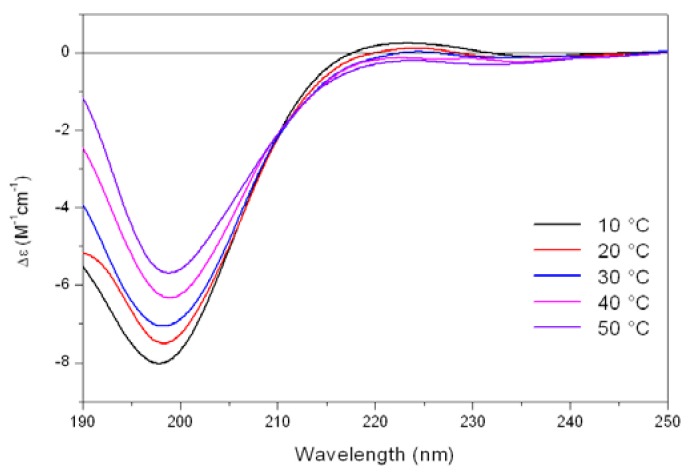
Far-UV CD spectra of Tat peptide (13.5 mM) in Tris-HCl buffer (5mM, pH 6.8) as a function of increasing temperature.

As shown in Figure 5, the CD spectrum of Tat at low temperature (10 °C) is characterized by a strong negative band at 196 nm and a weak positive band at about 223 nm. On raising the temperature, the 223 nm positive band disappears, meanwhile the absolute value of negative band at 196 nm decreases. In addition, an isodichroic point at 210-215 nm was observed permitting to describe this system as a combination of two different states, where the low-temperature form was best described by a PP_II_ like conformation.

The propensity of charged peptides to adopt an extended left-handed 3_1_-helix was initially proposed by Krimm and Mark [22] who demonstrated as electrostatic interactions would favor a helical rather than an unordered structure in ionized polypeptides. In these cases, when the steric energy is taken into consideration, the favored conformation is a left-handed helix having 2.5 - 3.0 residues per turn, where residues at positions *i* and *i+3* lie on the same edge of the helix. These results were confirmed by CD studies on ionized poly(Glu)_n_ or poly(Lys)_n_ by Holzwarth and Doty [23] which showed that the spectra of these compounds differ significantly from those characteristic of unordered polypeptides, while resembling that of the PP_II_ helix. In this way, the uncharged residues of Tat are located on the same edge of the helix, whereas the Arg and Lys residues are mostly located on the other two sides of the PP_II_ helix, and the adopted spatial disposition is suitable for the interaction with the cellular membrane representing the first step of the internalization process.

Ho *et al.* [24], using LINUS protein structure and GRASP molecular surface predictive programs, suggested that this peptide might have similarities with the amphipatic α-helix structure present in many plasma membrane fusing peptides, like toxins, and designed a potent cell penetrating peptide (33-fold increase in translocation) derived from Tat, containing only three arginine residues at the 4, 7 and 10 positions. In contrast with this finding, other groups reported lack of α-helicity for Tat as detected by CD measurements [11,25], confirming our results. 

In SDS micelles and in 9:1 v/v TFE/buffer, the CD spectra of kFGF and Nle^54^-Antp peptides (peptides **1** and **2**) were characterized by two negative bands at 222 and 208 nm, respectively, and a positive maximum at 193 nm, characteristic of the presence of α-helical structures. Also the Tat peptide (peptide **3**) adopted an α-helix conformation in 90% TFE solution, meanwhile in micellar SDS solution its behavior closely resembles that showed in aqueous solution: a left-handed PP_II_ like conformation at low temperature and an unordered conformation at room temperature (20). On the contrary, the CD spectra of oligoarginine peptides in both 90% TFE and in micellar SDS solution were characterized by a negative band at about 200 nm at all tested temperatures, characteristic of the irregular conformation of the peptide chain.

**Figure 6:**
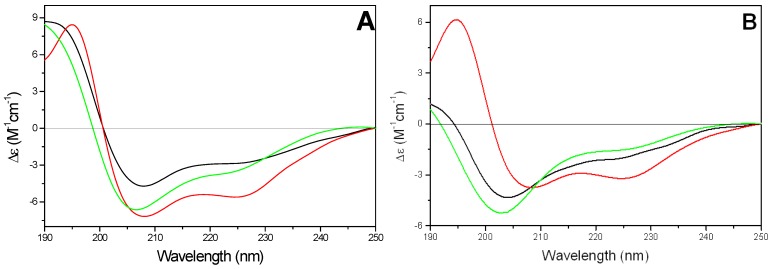
Far-UV CD spectra of kFGF (**1**) (11.9 µM) (A) and of kFGF-HS1pY (**1a**) (10.9 µM) (B) in presence of SUVs of different composition (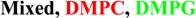).

In the presence of phospholipid SUVs, CPPs exhibited a conformational behavior completely different from that showed in membrane mimicking environment (SDS micelles or TFE). In particular, the Nle^54^-Antp peptide (peptide **2**) showed a largely random conformation in presence of zwitterionic DMPC vesicles, whereas in presence of anionic DMPG SUVs the peptide adopted mainly a β-sheet structure. In mixed DMPC/DMPG vesicles the CD spectrum strongly resembled that of an α-helix where the negative band is centered at 222 nm (data not shown). On the contrary, the kFGF peptide (peptide **1**) adopted mainly an α-helix conformation in all tested phospholipids (Figure 6A). The amount of α-helix conformation was not significantly affected by the change of temperature below and above lipid gel to liquid crystal phase transition (about 23 °C, data not shown). The estimated helicities were 35-40% in the presence of either DMPG or DMPC/DMPG vesicles and 55-60% in the presence of DMPC SUVs, respectively.

In the presence of zwitterionic or mixed vesicles the CD spectra of the Tat peptide were similar to those observed both in buffer and in micellar SDS solutions at room temperature. On the contrary, the CD pattern in presence of negatively charged DMPG vesicles closely resembled that obtained in 90% TFE solution, even if the negative band at 222 nm disappeared in a shoulder (data not shown).

The CD spectra of oligoarginines in presence of DMPC and mixed DMPC/DMPG SUVs were characterized by an evident positive band near 220 nm and a negative band at about 200 nm, which intensity decreased at the increasing of the percentage of negatively charged phospholipid. 

The conjugation with HS1pY peptide strongly affected the CD spectra of all CPPs, even if a rationalization of its effect is not possible. The characteristic α-helical conformation of the kFGF peptide (peptide **1**) decreased or disappeared. In particular, in the presence of negatively charged SUVs, the CD spectrum of peptide **1a** was characterized by a negative band at 202-203 nm with a shoulder at about 225 nm. The intensity of the negative band was strongly correlated to the charge density of SUV: it was maximum in the presence of DMPG vesicles and decreased with increasing the amount of DMPC phospholipids in the SUVs (Figure 6B).

A very similar behavior was observed for the Antp containing peptide **2a**. The elongation with HS1pY decreased the helical contribute both in micellar SDS and in 90% TFE solutions. In presence of zwitterionic DMPC vesicles the conjugated peptide adopts a largely irregular conformation, whereas the CD spectra in presence of DMPG or mixed vesicles closely resemble those described for the corresponding kFGF derivative.

The conjugation of HS1pY with Tat (peptide **3a**) stabilized the PP_II_ like conformation in buffer and in DMPC vesicles. On the contrary, the propensity to adopt an ordered structure in presence of negatively charged SUVs (DMPG or mixed vesicles) as well as in membrane mimicking environments (SDS and TFE) was strongly reduced, and the CD spectra are characterized by the presence of a negative band at about 200 nm. 

The introduction of the HS1pY peptide to the C-terminal end of oligoarginine peptides (peptides **4a – 6a**) induced very similar changes in the CD spectra recorded under different conditions. In buffer solution the CD spectra were characterized by the presence of two positive band at about 200 and 225 nm, meanwhile in micellar SDS solution the spectra showed a strong negative band at about 200 nm, with a shoulder at 225-230 nm. A very similar behavior was observable in the CD spectra in the presence of phospholipid vesicles. In particular, in the presence of DMPC or DMPG phospholipids the spectra were characterized by a positive band at 230 nm, suggesting the presence of a PP_II_ helix conformation. Surprisingly, the CD spectra of peptides **4a, 5a **and **6a** in TFE solution were characterized by a very low intensity of the dichroic signal and by the presence of negative band at 205 nm and positive band at about 190 nm.

### 2.4. Delivery of HS1pY peptide into mammalian cells

**Figure 7:**
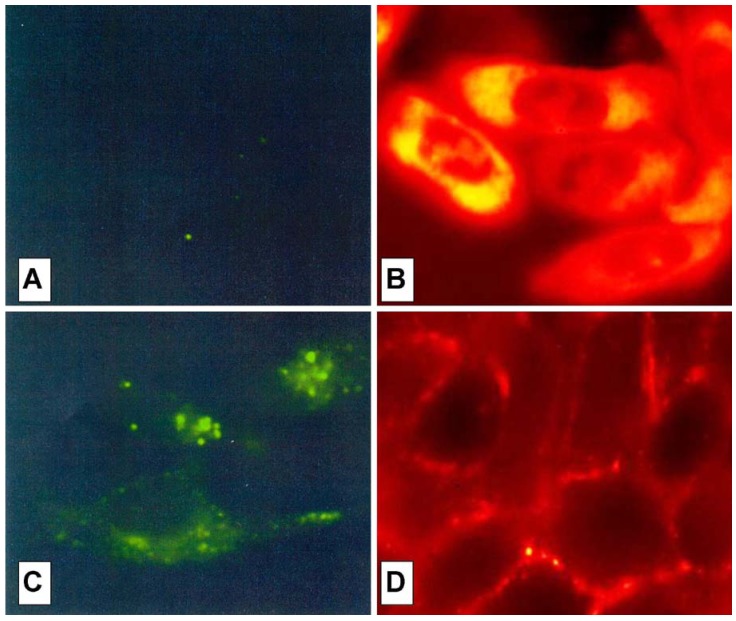
Confocal analysis of the HS1pY uptake. CHO cells were incubated at 4 °C (A) or 37 °C (B, C and D) for 30 min with 12.5 µM concentration of either the phosphodecapeptide (A and C) or the conjugated Tat (peptide **3a**) (B) and kFGF (peptide **1a**) (D). Preparation of the cells and details of the microscopy are described in the Experimental Section.

The cell peptide uptake was examined by confocal microscopy (Figure 7), using the carboxyfluoresceinyl labeled peptides. Briefly, the peptides were added (12.5 µM) to the incubation medium of CHO (Chinese hamster ovary) cells, for 30 min, 1 h and 2 h at 37 °C. Cells were washed and peptide internalization detected by confocal microscopy. In these conditions, previous detected membrane-induced carboxyfluorescein quenching (see fluorescence studies) did not affect in efficient way the cell peptide uptake detection.

Surprisingly, the conjugation of the HS1pY peptide destroyed the cell-penetrating properties of kFGF and R6 constructs (peptides **1a** and **4a**), meanwhile all the other constructs (peptides **2a**, **3a**, **5a** and **6a**) were internalized by cells. Maximal internalization was reached after 30 min; prolonged incubation times did not increase fluorescence visible inside cells (data not shown). Fluorescence was apparent throughout the cytoplasm and the nuclei of the cells and appeared to localize mainly to the nucleus. Works are in progress to identify the organelle(s) that is accessed with conjugated peptides.

Figure 7 also shows that, while the parent phospho-decapeptide (peptide **7**) could not penetrate cells at 4 °C (panel A), few fluorescence spots were visible inside control cells incubated at 37 °C, probably as a consequence of an endocytotic process (panel C).

The comparison of these data with the data of our biophysical investigation suggested that the single interaction with the cell membrane and its binding affinity, evaluated by fluorescence studies on vesicle models, per se do not confer specific translocation features, and more important, do not govern cell-penetrating capability.

Recent works [26,27] showed that the major route for Tat and Nle^54^-Antp peptide-mediated cellular uptake of cargo is endocytosis, rather than a translocation mechanism. In addition, reevaluation of the mechanism of internalization of cationic CPPs demonstrated that uptake was temperature-dependent indicative of a step requiring energy [27] as well as reported for signal peptide vectors (kFGF) [5,6]. Cationic peptides that initially accumulated on the cell surface in small patches (a process that was temperature-independent) were then internalized in intracellular vesicular structures. One can hypothesize that the high positive charges of conjugated peptides promote the binding to the cell surface. In addition, CPPs uptake by endocytosis rather than translocation mechanism may explain our difficulty to demonstrate the ability of these cationic peptides to pass across a non-cellular phospholipid bilayer (data not shown).

## 3. Experimental Section

### 3.1. Peptide Synthesis

Fmoc-protected amino acids and pre-loaded resins were purchased from Calbiochem-Novabiochem (Läufelfingen, Switzerland). Peptides were synthesized by solid-phase procedures on Wang resin using an Advanced Chemtech model 348Ω peptide synthesizer and the Fmoc chemistry in 0.05 mmolar scale. HBTU/HOBt activation employed a three-fold molar excess (0.2 mmol) of Fmoc-amino acids in DMF solution for each coupling cycle unless otherwise stated. Coupling time was 40 min. Deprotection was performed with 20% piperidine. Coupling yields were monitored on aliquots of peptide-resin either by the Kaiser test for the amino groups or by evaluation of Fmoc displacement [28]. To preclude side-reactions in the synthesis of the kFGF peptide a pre-loaded H-Pro-2Cl-Trytil resin was used instead of Wang resin [29]. 

Carboxyfluoresceinyl-labeled peptides were obtained by treatment of 0.025 mmol of H-peptides-resin with 0.1 mmol of (5)6-carboxyfluorescein *N*-succinimide ester (Fluka, Buchs, Switzerland) in presence of 0.2 mmol of DIEA in dry DMF, after elongation of peptide chains with a β-Ala residue.

Peptides were side-chain deprotected and removed from the resin by TFA treatment in the presence of 2.5% TIS, 2.0% anisole and 0.5% water, and then precipitated by addition of diethyl ether. Crude peptides were purified by preparative reversed-phase HPLC using a Shimadzu LC-8 (Shimazdu, Kyoto, Japan) system with a Vydac 218TP1010, 10µ, 250x10 mm column (Grace Davison Discovery Sciences, Deerfield, IL). The column was perfused at a flow rate of 12 mL/min with a mobile phase containing solvent A (0.05% TFA in water) and a linear gradient from 10% to 30% of solvent B (0.05% TFA in acetonitrile/water, 9:1 by vol.) in 40 min. The fractions containing the desired product were collected and lyophilized to constant weight in the presence of 0.01 N HCl. Analytical HPLC analyses were performed on a Shimadzu LC-10 instrument fitted with a Jupiter C18, 10 µ, 250 × 4.6 mm column (Phenomenex, Torrance, CA) using the described solvent system (solvents A and B), with a flow rate of 1 mL/min, and detection at 216 nm. All peptides showed less than 1% impurities. Molecular weights of compounds were determined by ESI-MS on a Mariner (PerSeptive Biosystem, Foster City, CA) mass spectrometer instrument. The mass was assigned using a mixture of neurotensin, angiotensin and bradykinin, at a concentration of 1 pmol/μL, as external standard. The amino acid compositions of the peptide acid hydrolysates (6 M HCl, 22 h at 110 °C in sealed evacuated vials) were determined with a Carlo Erba 3A30 (Milan, Italy) amino acid analyzer.

### 3.2. Vesicles Preparation.

SUVs were prepared as follows. Phospholipid (30 mg, about 43 µmol) was dissolved in *tert*-butanol and lyophilized for two days in a glass tube. The dried lipid was hydrated in 3 ml of 5 mM Tris-HCl buffer (pH 6.8) with repeated vortexed mixing at 40 °C for 30 min. The suspension was sonicated for 30 min (pulse mode: 2.5 sec pulse on, 1.0 sec pulse off) using a microprobe type sonicator (Vibra-Cell, Sonics & Materials, Inc., Newtown, CT, USA), stabilized for 90 min at 40 °C and then centrifuged (27,000*g*). Vesicle sizes were determined by dynamic light scattering using a Nicomp 370 autocorrelator equipped with a Spectra-Physics 2016 argon laser. The average hydrodynamic diameters were of 56 nm and 36 nm for DMPG and DMPC vesicles, respectively. These SUVs were used for the CD and fluorescence studies.

Dye-entrapped SUVs were prepared with a lipid film of desired composition by using 70 mM calcein in Tris-HCl buffer (Tris 20 mM, NaCl 150 mM, and EDTA 1 mM; pH 7.4) as a hydrating solution. Calcein-entrapped vesicles were separated from free calcein on a Sephadex G75 column (20 x 1.3 cm). Phospholipid concentration in dye-entrapped SUVs was determined as reported by Raheja *et al.* [30].

### 3.3. Circular Dichroism.

CD spectra were recorded using a nitrogen-flushed Jasco spectropolarimeter model J715 (Tokyo, Japan) using a 0.1 cm quartz cell. The CD spectra were recorded using a bandwidth of 2 nm, a scan speed of 10 nm/min and a time constant of 4 s. All spectra were recorded in the 5 mM Tris-HCl, pH 6.8 buffer. Each spectrum was the average of six scans with background of the buffer subtracted. Temperature was controlled at 25 °C by a Haake model F3 temperature controller. The helical content was determined using the CDPro software analysis program [31]. This program uses three methods for estimating protein secondary structure fractions from CD spectra. These methods are implemented in the software packages CONTIN/LL, SELCON3, and CDSSTR. Each of these three methods uses a different algorithm for analyzing a given protein CD spectrum, but they all use a single data-file structure [31].

### 3.4. Fluorescence spectroscopy.

Fluorescence emission spectra were recorded on a Perkin-Elmer fluorescence spectrophotometer LS-50B (Norwalk, CT) using emission and excitation slit widths of 5 nm at 25 °C under constant magnetic stirring, subtracting the buffer background and correcting for dilution. Excitation wavelengths were at 485 nm for carboxyfluoresceinyl moiety and the emission spectra were recorded from 500 to 640 nm. Absorbance for all solutions at the excitation and emission wavelengths was less than 0.05 units to minimize inner-filter effects.

Spectroscopic titrations of peptides with SUVs were performed as reported by Surewicz and Epand [32]. Appropriate aliquots of DMPC, DMPG or DMPC-DMPG (3:1 molar ratio) liposomes were successively added to a solution (2.5 ml) of peptides in Tris-HCl buffer (5 mM, pH 6.8). After each addition of liposomes, the mixture was kept at 25 °C for 5 min to achieve the equilibrium. The binding isotherms were analyzed as a partition equilibrium using the following formula:

X_b_ = K_p_C_f_

where X_b_ is defined as the molar ratio of bound peptide per total lipid, K_p_ corresponds to the partition coefficients, and C_f_ represents the equilibrium concentrations of the free peptide in the solution. The curve resulting from plotting X_b_ versus the free peptide concentration, C_f_, is referred to the conventional isotherm. To calculate X_b_, the fraction of the membrane bound peptide f_b_ is determined with the following formula:

f_b_ = (F-F_0_) / (F_∞_-F_0_)

where F_0_ is the fluorescence of the unbound peptide, F the fluorescence of the bound peptide, and F_∞_ the fluorescence signal obtained when all the peptide is bound to lipids. In several cases, the plateau level reached during titration was taken as F_∞_, but in those instances where no plateau was achieved, a value for F_∞_ was extrapolated from a double reciprocal plot of F (total peptide fluorescence) versus C_L_ (total concentration of lipids), as previously suggested by Schwarz [33]. With f_b_ known, the C_f_ value as well as the extent of peptide binding (X_b_) could be calculated. Partition coefficients were calculated from the initial slope of each conventional isotherm (for details of the calculation, see [33,34]).

The release of calcein from SUVs was monitored by fluorescence at an emission wavelength of 520 nm (excitation wavelength of 490 nm). The maximum fluorescence intensity corresponding to 100% leakage was determined by addition of 10 μL of 10% Triton X-100 to the sample (3 ml). The apparent percent leakage was determined according the following equation:

% = 100•((F-F_0_)/(F_max_-F_0_))

where F and F_max_ correspond to the fluorescence intensity before and after the addition of detergent, respectively. F_0_ represent the fluorescence of intact vesicles. Peptide concentrations were determined either by absorption spectroscopy (ε_485nm_= 80,000 cm^-1^M^-1^) or by the average amino acid recovery from acid hydrolyzate of an aliquot of the peptide solutions.

### 3.5. pI determination

The p*I* values were calculated using the Demo version of Protein Tools™ (ChemSW, Fairfield, CA, USA). The pK_a_ values for the acidic amino acid residues were: 4.0 for Asp, 4.4 for Glu, 10.0 for Tyr and 3.5 for the C-terminal carboxylate. For the basic residues, pK_a_ values used were: 12.0 for Arg, 10.0 for Lys and 8.0 for the N-terminal amine. For the phosphate group attached to tyrosine, pK_a1_ of 1.0 and pK_a2_ of 6.1 were used [35]. 

### 3.6. Cell Penetration Assay.

CHO cells were seeded into glass coverslips at 30,000 cells/well in Ham's F12 medium with 10% fetal calf serum and antibiotics. After overnight incubation, phospho-peptides were prepared as dilution series in cell medium (12.5, 25 and 50 µM) and were added to cells. At the end of the incubation (30 min, 1 h and 4 h) at both 4 °C and 37 °C, cells were rinsed with PBS three times and fixed for 30 min at 4 °C in formaldehyde (4% in PBS), rinsed again and treated with 50 mM NH_4_Cl. Coverslips were then mounted in glycerol for observation of cells by fluorescence microscopy. Fluorescence microscopy was carried out using a Zeissew Axiovert 100, equipped with a 12 bit digital CCD videocamera Micromax (Princeton Instruments, Trenton, NJ, USA). Data were acquired and analyzed by Metamorph software (Universal Imaging). 

## 4. Conclusions

The potential application of CPP-mediated transport in vivo necessitates knowledge about the mechanism of transport and the influence of cargoes on the cellular uptake of particular CPPs. CD and fluorescence spectroscopies furnished useful information about the secondary structure of both free and conjugated CPPs in membrane-mimicking environments and their interaction with phospholipid vesicles, respectively, yielding useful enlightenment on the initial steps of CPP-mediated cellular uptake, which has been postulated to be the interaction of CPP with lipids in the plasma membrane. As shown in Figure 3, with the exception of R10 derivative, CPPs and their HS1pY conjugates are characterized by an evidently different affinity towards phospholipids that may be explained invoking the electrostatic repulsion between negatively charged SUV and HS1pY cargo. In a previous work, Jiang *et al.* [36] used an anionic peptide (six to nine consecutive Glu residues) to block the cellular uptake of a polycationic CPP (9 Arg residues) by intramolecular electrostatic interaction. To realize this inhibition the authors introduced between the CPP and the polyanionic peptide a cleavable sequence that can adopt a turn conformation. The absence in our conjugates of a suitable sequence minimizes or does not permit the realization of these intramolecular interactions. On the contrary, intermolecular interactions, as demonstrated by Jiang *et al.* [36], are indifferent in the inhibition of the cellular uptake, and so also in modify the affinity of CPP-conjugates towards SUVs. Cellular uptake experiments showed that not all tested CPP-HS1pY constructs have been internalized inside cells, even if interacted with phospholipids vesicles.

Therefore, even if certain physico-chemical properties (conformation, positive charge, hydrophobicity, and amphipathicity) may govern the initial step of the cellular uptake of these peptides, our data suggest that there is not any correlation with the uptake properties of the peptides in the cell line tested, indicating that other properties, in addition to lipid binding, might be involved in the initial binding to the membrane and subsequent translocation process of CPPs.

## Abbreviations

DIEA: *N,N*-diisopropylethylamine
DMF: *N,N*-dimethylformamide
DMPC: 1,2-dimyristoyl-rac-glycero-3-phosphocholine
DMPG: 1,2-dimyristoyl-sn-glycero-3-phospho-rac-(1-glycerol)
EDTA: ethylenediaminetetraacetic acid
Fmoc: 9*H*-fluoren-9-ylmethoxycarbonyl
HBTU: O-(benzotriazol-1-yl)-*N,N,N’,N’*-tetramethyl-uronium hexafluorophosphate
HOBt: 1-Hydroxybenzotriazole
SDS: sodium dodecyl sulfate
TFA: trifluoroacetic acid
TFE: 2,2,2-trifluoroethanol
TIS: triisopropylsilane
Tris: tris(hydroxymethyl) aminomethane
